# Occupational rhinitis 

**DOI:** 10.5414/ALX02165E

**Published:** 2021-01-22

**Authors:** Sebastian Kotz, Lisa Pechtold, Rudolf A. Jörres, Dennis Nowak, Adam M. Chaker

**Affiliations:** 1 Department of Otolaryngology – Head and Neck Surgery, Klinikum rechts der Isar, Technical University of Munich, Munich, Germany,; 2 Center of Allergy and Environment (ZAUM) of the Technical University of Munich (TUM) and the Helmholtz Zentrum München (HMGU), and; 3 Institute and Outpatient Clinic for Occupational, Social and Environmental Medicine, Clinical Center of the Ludwig Maximilian University Munich, Germany, Comprehensive Pneumology Center (CPC) Munich, German Center for Lung Research (DZL)

**Keywords:** allergic, non-allergic occupational rhinitis (OR), occupational asthma (OA), occupational disease, BK 4301, nasal provocation test, work-related-rhinitis, work-exacerbated rhinitis

## Abstract

Occupational rhinitis (OR) has so far received little attention even though it shares common pathophysiological features and trigger factors and is closely associated with occupational asthma (OA). Work-related exposure to certain substances, such as animal dander, is considered to be the main factor for the development of OR. The new EAACI definition of OR stresses the causal relationship between workplace exposure and onset of rhinitis symptoms as opposed to previous definitions that mainly focused on a temporal relationship between workplace exposure and occurrence of nasal symptoms. Also, it has been suggested to use the term “work-related rhinitis” for classifying the different forms of rhinitis associated with the workplace. These forms can be subdivided into allergic or non-allergic OR, which is due to causes and conditions related to a particular work environment, as well as work-exacerbated rhinitis, which is defined as a pre-existing rhinitis exacerbated by exposure at the workplace. Even though taking a detailed patient history is especially important when it comes to diagnosing OR, the gold standard for confirming the diagnosis is nasal provocation testing. Best possible symptomatic relief and prevention of development of OA constitute the main therapeutic objectives in OR. Treatment options consist of total avoidance of trigger substances (main goal), reduction of exposure to certain substances, and pharmacotherapy. Furthermore, it is important to note that allergic OR is an occupational disease in Germany (Berufskrankheit No 4301) and needs to be reported to health authorities.

**German version published in Allergologie, Vol. 43, No. 6/2020, pp. 239-246.**

## Introduction 

The improved understanding of the interactions between the upper and lower airways has given increasing importance to the clinical features of rhinitis [1]. It has also been shown that occupational asthma (OA) and occupational rhinitis (OR) are closely related and share many causative factors [2]. Accordingly, a high prevalence of rhinitis symptoms in patients with occupational asthma has been reported [3]. However, OR has received a lot less attention than OA [4]. Therefore, the socioeconomic impact of OR remains significantly underestimated compared to OA [5]. 

Occupational exposure to certain substances, such as flour dust or the epithelia of laboratory animals, is considered one of the main causes of OR [6]. However, the epidemiological associations that contribute to the development of OR have not yet been clarified in desirable detail. A Finnish study has described that the risk of developing OR is particularly high for certain occupational groups such as bakers, food processing workers, farmers, veterinarians, animal breeders, electronic product manufacturers, and boat builders [7]. However, the incidence of OR in the general population still remains largely unknown [8]. 

Exposure to certain substances, nicotine abuse, and the presence of atopic predisposition have been considered major risk factors for the development of OR [9]. A dose-dependent association between exposure and IgE-mediated sensitization has been shown for several substances (e.g., flour dust) [10]. Atopic predisposition is also known to be associated with an increased risk of sensitization to various substances and, as a consequence, with OR caused by these substances [11]. However, despite intensive efforts, no clear link between nicotine abuse and OR has yet been identified [12]. 

The definitions of OR used so far are mainly based on a temporal link between workplace exposure and the occurrence of nasal symptoms (e.g., nasal breathing obstruction, rhinorrhea) [9]. Due to the similarities and interactions between the pathomechanisms of rhinitis and asthma, a common definition of OA and OR seems to be very useful [13]. Therefore, the European Academy of Allergy and Clinical Immunology has developed the following definition of OR: “Occupational rhinitis is an inflammatory disease of the nose characterized by intermittent or persistent symptoms such as nasal obstruction, rhinorrhea, sneezing and itching. In addition, it is associated with a variable degree of obstruction of nasal airflow and/or the occurrence of nasal hypersecretion. It is caused by factors that can be attributed to a particular workplace environment and is not associated with factors that occur outside the workplace” [14]. The causal and not only temporal relationship between workplace exposure and the occurrence of the disease, as set out in this definition, is crucial. 

However, there is growing evidence that workplace exposure to certain substances can cause or aggravate different forms of rhinitis [15]. Therefore, it is recommended that the term “work-related rhinitis” be used for the various classifications of rhinitis associated with the workplace environment. The sub-categories of work-related rhinitis can then be differentiated according to the underlying pathomechanisms and different clinical manifestations. Consequently, OR describes a form of rhinitis caused by exposure to certain substances in the workplace environment. Work-exacerbated rhinitis is a form of rhinitis caused by exposure to certain substances in the workplace, whereas the symptoms of pre-existing allergic or non-allergic rhinitis are aggravated by workplace exposure, whereas the disease itself is not caused by this occupational exposure [16]. 

OR can be divided into an allergic and a non-allergic form. The allergic form of OR is characterized by the occurrence of nasal hyper-reactivity to specific workplace substances. Increased nasal reactivity occurs only after an initial latency period in which sensitization to the triggering substance occurred. Repeated exposure to the triggering substance can then lead to intermittent as well as persistent nasal symptoms. The form of allergic OR can be triggered either by IgE-mediated (e.g., animal epithelia) or non-IgE-mediated reactions (e.g., isocyanates) in which certain substances act as haptens. In contrast, the non-allergic form of OR is triggered by irritative, non-immunological mechanisms and has no latency period before manifestation of the first clinical symptoms [17]. 

**Figure 1:**
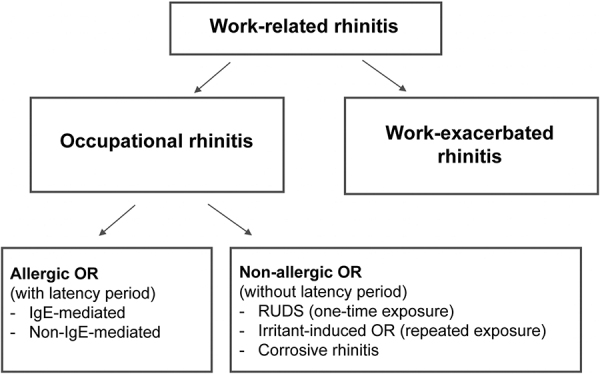
Overview of classifications of occupational rhinitis, work-related rhinitis, and work-exacerbated rhinitis, modified from Shao and Bernstein [20].

The non-allergic form of OR also comprises different subgroups. If a single exposure to a high concentration of irritant substances (e.g., chlorine) leads to symptoms, it is called reactive upper airways dysfunction syndrome (RUDS) [18]. If the symptoms are only caused after multiple exposures to irritant substances (e.g., formaldehyde), this is called irritant-induced OR. The most pronounced form of non-allergic, OR is represented by “corrosive rhinitis”. This can lead to persistent nasal mucosal inflammation and, as a consequence, even to ulceration and perforation of the nasal septum [19]. Since the clinical appearance of work-related rhinitis is very similar to the appearance of OR, the diagnosis of workplace-exacerbated rhinitis should be made only after thorough diagnostic testing has ruled out sensitization to workplace-specific substances. The classifications of the different groups of workplace-exacerbated rhinitis are illustrated in Figure 1. 

The diagnostic workup for OR should include both the symptoms of rhinitis and the association of these symptoms with the workplace environment. Since the diagnosis of OR can have serious social and financial consequences, objective methods should be used to avoid misclassification of patients. In addition to the diagnosis of OR, a possible involvement of the lower respiratory tract should always be investigated, the assessment of which may include questionnaires, spirometry, and measurement of exhaled nitric oxide (NO) [21]. 

Accurate anamnesis plays a key role in the diagnosis of OR. In addition to evaluating the severity of the symptoms and their impact on the patient’s quality of life, special attention should be paid to current tasks in the workplace, processes in adjacent work areas, recent changes in materials used or steps performed, and the hygienic conditions in the workplace. One of the main objectives of the anamnesis is to determine the temporal connection between the onset of rhinitis symptoms and occupational exposure. Therefore, special attention should be paid to the length of employment prior to the onset of the nasal symptoms (latency period). It should also be determined whether exposure to certain substances or the performance of certain work steps is associated with the onset or worsening of clinical symptoms. Furthermore, it is of interest whether an improvement of symptoms occurs when working at a distance from the workplace environment (e.g., on weekends, vacations). 

Although taking a medical history of suspected OR is an essential step in the diagnostic process, it alone is not specific enough to make a diagnosis of OR [22]. With the help of anterior rhinoscopy or nasal endoscopy, the macroscopic appearance of the nasal mucosa can be assessed directly and, in addition, the presence of other rhinological pathologies (e.g., nasal polyps) that could be differentially responsible for the clinical symptoms can be excluded. The performance of a rhinomanometric examination also makes it possible to objectify the nasal patency or the airflow through the nose [23]. In addition, rhinomanometry is an excellent tool for recording the results of a nasal provocation test. By obtaining cytological samples from the nose (nasal secretions and biopsies), inflammatory cells and their mediators can be directly quantified [24]. While biopsy collection is often of limited use due to its invasive nature, nasal curettage allows relatively simple and painless cytological sample collection. It can be assumed that the collection of nasal secretions will play an essential role in the diagnosis of OR as soon as this diagnostic tool will become more easily available. Immunological tests, such as the skin prick test or serological tests for the detection of allergen-specific IgE antibodies, can also be used for the investigation of IgE-mediated sensitization to substances occurring at the workplace. However, the applicability of immunological tests is currently limited by the low availability of commercially available test substances. 

Furthermore, it should be emphasized that even asymptomatic persons who were exposed to a certain substance can show a positive test result in immunological tests. However, a negative immunological test result against the corresponding potential allergens makes the diagnosis of OR in relation to a specific agent unlikely [25]. The performance of a nasal provocation test remains the gold standard for diagnosis of OR [26]. Provocation testing can objectify and document the causal relationship between exposure to a specific agent and the occurrence of symptoms of rhinitis. A differentiation between irritant and specific allergic mechanisms must be made depending on the triggering agent and the reaction. 

## Local rhinitis 

It is ultimately unclear what quantitative role a purely local allergy plays in (occupational) allergic rhinitis. It is a phenomenon in which the clinical manifestation corresponds to allergic rhinitis, but prick tests and specific IgE determinations in serum are negative and nasal provocation tests are positive [27]. This phenomenon is reported with a prevalence of between 20 and 30% of all rhinitis patients [28]. The existence of this phenomenon should be a reason to carry out nasal provocation tests with the suspected/accused allergen if the patient’s medical history is positive, the prick test negative, and the specific IgE determination negative – if in doubt, once too often rather than not enough. 

## Therapy 

The treatment of OR has two goals. Firstly, the minimization of the clinical symptoms of rhinitis and its impact on the patient’s quality of life, secondly, the prevention of the development of OA. Treatment options include avoidance of trigger factors, exposure reduction, and pharmacological therapy [29]. 

In principle, the primary goal of an intervention should be the complete avoidance of the triggering substances. However, in order to achieve complete absence of exposure, it is usually necessary to make drastic changes in occupational activities, often with serious social and financial consequences [30]. Therefore, as an alternative to a complete avoidance of exposure, there are various ways to reduce exposure to certain substances, such as wearing protective equipment at the workplace, reducing the exposure time or changing the materials used or the work steps to be performed [31]. However, it should be noted that if the interventions are primarily aimed at reducing exposure rather than at complete avoidance of triggering substances, regular clinical follow-up of rhinitis symptoms is required. In addition, special attention should be paid to a possible initial manifestation of the symptoms of OA. 

Drug treatment options for OR are consistent with the treatment recommendations for non-OR as outlined in the current guidelines. 

Symptomatic treatment with intra-nasal corticosteroids and antihistamines, as well as systemic antihistamines, are among the options available [32]. Although specific immunotherapy against certain occupational allergens (e.g., flour extracts, natural latex) has shown good response in certain studies, its broad clinical applicability is currently very limited due to the limited availability of standardized allergen extracts for many occupational substances [33]. Thus, it is unfortunately not to be expected that specific immunotherapy will be increasingly applied by certain occupational groups, such as veterinarians, in the future. 

However, drug therapy of OR should not be preferred to the avoidance of exposure, since the triggering substances of OR can often also lead to the manifestation of OA. Due to the interaction of the upper and lower respiratory tract, diagnosis and treatment of OR requires close cooperation between general practitioners, occupational physicians, ENT specialists, and pneumologists. 

Failure to diagnose or treat OR can lead to invalidity, severe comorbidities, and a significant socioeconomic burden on the patient and the health care system. Therefore, both employers and employees should be informed about the clinical manifestations of this occupational disease, its consequences and, in particular, possible options for prevention. Occupational medicine has a special role to play in reliably identifying the symptoms of occupational diseases. Likewise, regular occupational medical examinations contribute to the development of efficient prevention strategies and should be carried out as early as possible in the event of potential exposure. 

## Rhinitis as an occupational disease 

Work-related rhinitis can be an occupational disease. However, non-allergic rhinitis is not an occupational disease under German law. It can indicate inadequate working hygiene conditions. It is important to distinguish it from local allergic rhinitis, which can be an occupational disease (as defined by BK 4301). In order to distinguish one from the other, there is no getting around nasal provocation testing with the suspected allergen. Nasal provocation tests with irritants, however, make little sense. 

A well-founded suspicion of occupational allergic rhinitis must be reported. The addressee of the notification is the responsible insurance institution or the medical inspector of labor [34]. The legal definition of occupational disease 4301 reads: “Obstructive respiratory diseases (including rhinopathy) caused by allergenic substances that have forced people to refrain from all activities that were or could be the cause of the development, aggravation or resurgence of the disease”. In the case of occupational disease 4302, defined as obstructive respiratory diseases caused by chemical-irritant or toxic substances, rhinitis is not included. 

The sense of a suspected occupational disease notification – apart from the legal obligation to notify – results mainly from the fact that the institutions of the state accident insurance can make effective preventive measures in the sense of § 3 of the Ordinance on Occupational Diseases: This § 3 of the Ordinance on Occupational Diseases regulates measures to prevent the occurrence of occupational diseases and transitional benefits. It reads: “If there is a risk of an occupational disease arising, recurring, or worsening for insured persons, the insurance institutions must counteract this risk by all appropriate means. If the danger cannot be eliminated, the insurance institutions must work towards ensuring that the insured persons refrain from the dangerous activity. The authorities responsible for medical occupational health and safety must be given the opportunity to make a statement. Such danger exists if the risk of injury to the insured person at the specific workplace exceeds the degree that exists for other insured persons in a comparable occupation. There must therefore be a concrete individual risk for the insured person. 

Preventive measures are for example:

- technical and organizational measures (e.g., replacement of hazardous working materials, e.g., replacement of highly dusty flours with less dusty ones);
- personal protective measures (for example, fan-assisted respiratory protection);
- medical measures (outpatient/inpatient treatment, special therapeutic measures).

The spectrum of § 3 measures is very broad: It reaches from the offer of a respiratory consultation up to conversion measures in the company, for example, if because of threatening occupational illness feeding must be changed from hay to silage.

- Of the current 80 occupational diseases, 9 contain a compulsory injunction, i.e., the addition “that have forced the farmer to refrain from all activities that were or could be the cause of the development, aggravation, or resurgence of the disease”. These 9 occupational diseases – including the occupational disease 4301 relevant to occupational allergic rhinitis in the sense of OR – currently account for 50% of all suspected disease reports. This “obligation to refrain”, which has medically and legally not been uncontroversial for quite some time, was primarily intended to
- exclude minor cases of illness from recognition as a BK,
- to prevent further health hazards when the same insured activity is continued to be carried out as it was before, by giving up this activity, if these health hazards cannot be avoided by prevention measures deployed by the company.

On January 1, 2021, a legal reform comes into force that removes the obligation to cease and desist. According to the assessment of the statutory accident insurance and the legislator, the objectives pursued to date with the cease-and-desist order can be achieved with other regulations, and in some cases even more precisely. In any case, the primary goal remains the avoidance of aggravation of diseases in individual cases. To this end, it is essential to intensify prevention activities and the active participation of those affected. For the delimitation of bagatelle illnesses, now a specification of the existing occupational disease is necessary. In the future, too, an expert will have to assess in each individual case as to whether prevention measures are responsible for continuing the activity or whether it is advisable to give up the activity, so that occupational participation benefits (e.g., retraining at the expense of the accident insurance institution) can be considered [34]. 

## Funding 

No funding was received for this article. 

## Conflict of interest 

The authors do not indicate any existing conflict of interest.
